# Combination of STING agonist with anti-vascular RGD-(KLAKLAK)_2_ peptide as a novel anti-tumor therapy

**DOI:** 10.1007/s00262-024-03732-3

**Published:** 2024-06-04

**Authors:** Justyna Czapla, Alina Drzyzga, Joanna Ciepła, Sybilla Matuszczak, Magdalena Jarosz-Biej, Ewelina Pilny, Tomasz Cichoń, Ryszard Smolarczyk

**Affiliations:** Center for Translational Research and Molecular Biology of Cancer, Maria Sklodowska-Curie National Research Institute of Oncology, Gliwice, Poland

**Keywords:** cGAS-STING pathway, Anti-vascular therapy, RGD-based (KLAKLAK)_2_ peptide, α_v_β_3_ Integrin, Anti-cancer therapy

## Abstract

Immunotherapy is one of the most promising anti-cancer treatment. It involves activating the host's own immune system to eliminate cancer cells. Activation of cGAS-STING pathway is promising therapeutic approach for cancer immunotherapy. However, in human clinical trials, targeting cGAS-STING pathway results in insufficient or unsustainable anti-tumor response. To enhance its effectiveness, combination with other anti-cancer therapies seems essential to achieve synergistic systemic anti-tumor response.

The aim of this study was to evaluate whether the combination of STING agonist-cGAMP with anti-vascular RGD-(KLAKLAK)_2_ peptide results in a better anti-tumor response in poorly immunogenic tumors with various STING protein and α_v_β_3_ integrin status.

Combination therapy inhibited growth of murine breast carcinoma more effectively than melanoma. In melanoma, the administration of STING agonist alone was sufficient to obtain a satisfactory therapeutic effect. In both tumor models we have noted stimulation of innate immune response following cGAMP administration alone or in combination. The largest population of immune cells infiltrating the TME after therapy were activated NK cells. Increased infiltration of cytotoxic CD8^+^ T lymphocytes within the TME was only observed in melanoma tumors. However, they also expressed the “exhaustion” PD-1 receptor. In contrast, in breast carcinoma tumors each therapy caused the drop in the number of infiltrating CD8^+^ T cells.

The obtained results indicate an additional therapeutic benefit from combining STING agonist with an anti-vascular agent. However, this effect depends on the type of tumor, the status of its microenvironment and the expression of specific proteins such as STING and α_v_β_3_ family integrin.

## Introduction

Immunotherapy has emerged as one of the most promising cancer treatments. Its assumption is to reactivate, modulate and strengthen body’s own immune system to eliminate tumor cells. Immunotherapy uses cytokines, chemokines, and immune cells to reshape the tumor microenvironment (TME), leading to potent anti-tumor effects as well as prevents tumor recurrence. Cancer immunotherapy methods include, among others: monoclonal antibodies (checkpoint mAbs, therapeutic mAbs), small molecule drugs (targeting PD-1/PD-L1 axis, STING agonists, CXCRs inhibitors), adoptive cell therapy (CAR-T, TCR-T, TILs, CAR-NK) oncolytic viruses (adenoviruses, HSV-1) and cancer vaccines (dendritic cells stimulated ex vivo) [1]. Compared to standard cancer treatments (including chemotherapy, radiotherapy, and surgery), cancer immunotherapy results in significant improvements for patients in terms of progression-free survival (PFS), overall survival (OS) and quality of life. Therefore, immunotherapy has become the first line treatment for numerous cancer types. Although effective immunotherapies have been reported, statistically only a small minority (20–40%) of patients benefit from them [2]. The major limitations are immune-related adverse events, cytokine storm, patients low response rate or acquired resistance mechanisms. Therefore, further improvements for cancer immunotherapies are crucially needed. One of the immunotherapy methods, intensively developed in recent years, is activation of cGAS-STING pathway. It acts as innate immunity activator, sensing cytosolic DNA and inducing expression of genes encoding type I interferons and pro-inflammatory cytokines via the transcription factors IRF3 and NF-κB, respectively. Several types of STING agonists have been found, divided into three categories: (1) cyclic dinucleotides (CDNs, including cGAMP), (2) flavonoids (DMXAA and its analogs), (3) small molecule agonists. STING stimulators have been tested in preclinical models and some in clinical trials and have shown reduced tumor growth and tumor clearance capability [3]. However, in many refractory tumor models, targeting STING protein results in insufficient or unsustainable anti-tumor response. To enhance the effectiveness of cancer immunotherapy, combination of various therapeutic strategies seems essential to achieve synergistic systemic anti-tumor response.

Targeting tumor vasculature effectively reduces tumor burden. There are two types of agents targeting tumor vasculature: inhibitors of new capillaries formation (anti-angiogenic agents, AAs) or agents that destroy existing tumor blood vessels (vascular disrupting agents, VDAs). Destruction of endothelial cells leads to vascular system disruption and tumor cell necrosis through inhibition of oxygen and nutrition supply. VDAs divide into three groups: (1) Microtubule destabilizing drugs (combretastatins e.g. CA4P), (2) Flavonoids with anti-vascular functions (DMXAA), (3) Drugs targeting endothelial cells receptors (e.g. peptide RGD-(KLAKLAK_2_)) [4]. Most drugs in the latter group target receptors that are overexpressed on tumor endothelial cells, like α_v_β_3_ integrin or VEGF receptor. Their ligands are tripeptide Arg-Gly-Asp (RGD) or VEGF protein, respectively. These agents are composed of cognitive subunit and effector-toxin domain that after internalization effectively kill target cells. One example is the construct of RGD domain with proapoptotic peptide (KLAKLAK)_2_ that specifically binds to α_v_β_3_ integrin. Once bound, the peptide internalizes inside the cell, where the cytotoxic domain (KLAKLAK)_2_ causes binding and destruction of the mitochondrial membrane, leading to death of endothelial cells. Areas of tumor necrosis appear around the damaged vessels, and tumor shrinkage is observed [4].

The aim of this study was to evaluate whether the combination of immunotherapy activating the cGAS-STING pathway with the anti-vascular agent RGD-(KLAKLAK)_2_ will result in a better anti-tumor response in tumors with low level of STING protein (4T1 breast carcinoma) compared to tumors with high STING expression (B16-F10 melanoma). The study presents the influence of the tumor microenvironment on the results of anticancer therapy.

## Results

### Integrin β_3_ expression in breast carcinoma (4T1) and melanoma (B16-F10)

**Fig. 1:**
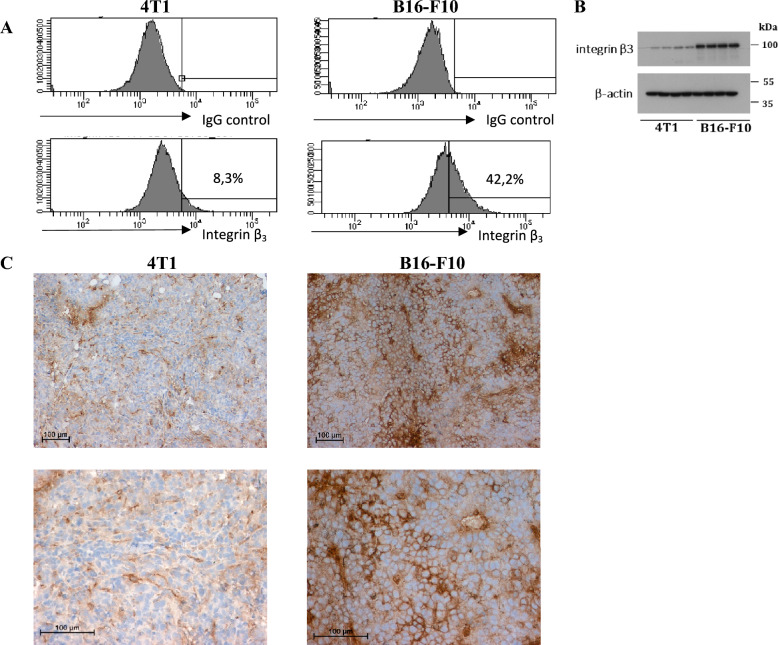
Integrin β_3_ subunit expression in murine breast carcinoma (4T1) and melanoma (B16-F10). **A** The expression of integrin β_3_ on 4T1 and B16-F10 cell lines was determined by flow cytometry. **B** Total protein was extracted from 4T1 and B16-F10 cell lines, and the level of integrin β_3_ was detected by western blotting. β-actin was used as a loading control, *n* = 4. **C** Immunohistochemical analysis was performed to visualize integrin β_3_ expression pattern in 4T1 and B16-F10 tumors, magnification 20x and 40x

In order to evaluate the possibility of RGD-(KLAKLAK)_2_ peptide targeting to α_v_β_3_ integrin receptors we identified β_3_ integrin subunit in 4T1 and B16-F10 cancer cell lines and in its corresponding tumors. We have shown that β3 subunit of the receptor is expressed by almost 50% of B16-F10 cancer cells and its strong expression in melanoma is demonstrated by cancer cells and endothelial cells (Fig. 1A, B, C). In 4T1 cells expression of β_3_ integrin is much lower (Fig. 1A, B). Similarly in breast carcinoma tumors, expression of β3 integrin in tumor cells is faint and is rather limited to blood vessels and stromal cells (Fig. 1C).

We assessed the level of STING protein in our previous study [5] and have shown that B16-F10 cells express elevated level of STING protein whereas 4T1 cells only trace amounts. Likewise, in 4T1 tumors STING protein was expressed mostly by cells of tumor stroma (fibroblast-like cells) and endothelial cells. In B16-F10 tumors, STING protein, apart from cancer-related cells, was abundantly expressed by cancer cells [5].

### Tumor growth inhibition following combination therapy

**Fig. 2:**
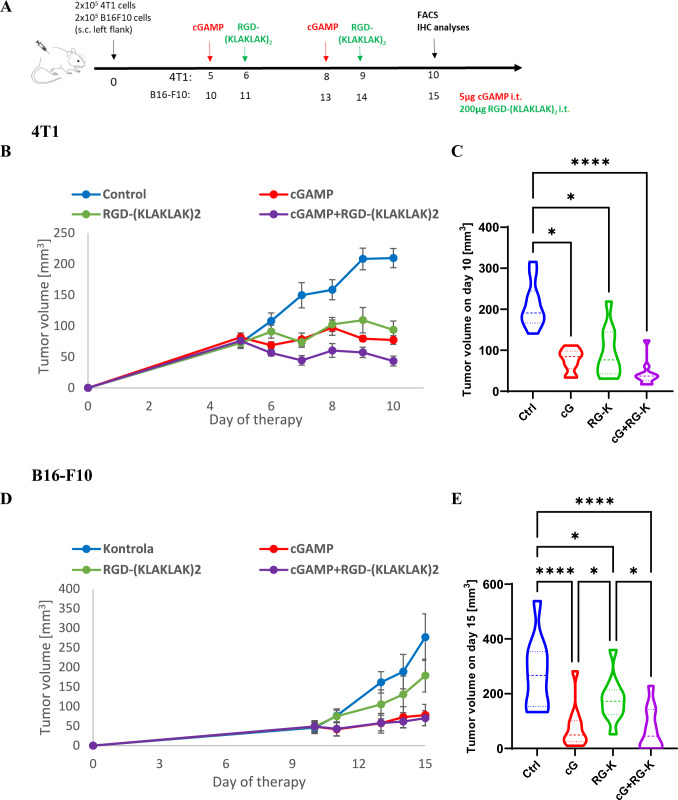
Inhibition of murine breast carcinoma (4T1) and melanoma (B16-F10) growth using combination therapy of cGAMP and RGD-(KLAKLAK)_2_. **A** Diagram depicting the treatment schedule in subcutaneous (s.c.) breast tumor of BALB/c mice and melanoma of C57BL/6NCrl mice. Red arrows indicate intratumoral (i.t.) cGAMP administration, green arrows intratumoral (i.t.) RGD-(KLAKLAK)_2_ administration. **B**, **D** Tumor volume was measured with a caliper every 1 or 2 days (mean ± SEM). **C**, **E** Diagram showing tumor volume on the last day of experiment. Data are representative of two independent experiments, *n* = 5 in each group. Statistical analysis was performed on the last day of experiment **C** ANOVA with post-hoc Fisher’s LSD test, **E** Kruskal–Wallis test with Dunn's multiple comparison post hoc test. *p < 0.05 ****p < 0.0001

We have evaluated the effect of STING agonist-cGAMP and anti-vascular peptide RGD-(KLAKLAK)_2_ on tumor growth in a single agent administration as well as in their combination. Mice with 4T1 tumors were treated with cGAMP and/or RGD-(KLAKLAK)_2_ as shown in the diagram of Fig. 2A. Each of the therapeutic agents used as monotherapy inhibited tumor growth. Tumor volume in both monotherapies was 2-times smaller than in control group and on the tenth day similar to the initial volume of the tumors at the beginning of therapy. Combination of these two agents resulted in moderate tumor growth inhibition (tumors were almost 5-times smaller compared to control: 43mm^3^ vs 210mm^3^ and about 1.5-times smaller than in both monotherapies) (Fig. 2B, C). Mice with B16-F10 melanoma were treated in the same way (Fig. 2A). B16-F10 tumors responded better to cGAMP treatment. However, there was no difference in tumor volume following cGAMP administration either as monotherapy or in combination with anti-vascular agent (tumors were almost 4-times smaller compared to control: 70mm^3^ vs 276mm^3^) (Fig. 2D, E). The administration of RGD-(KLAKLAK)_2_ resulted in 1.5-fold reduction in tumor volume compared to control.

### The effect of combination therapy on tumor tissue structure

**Fig. 3:**
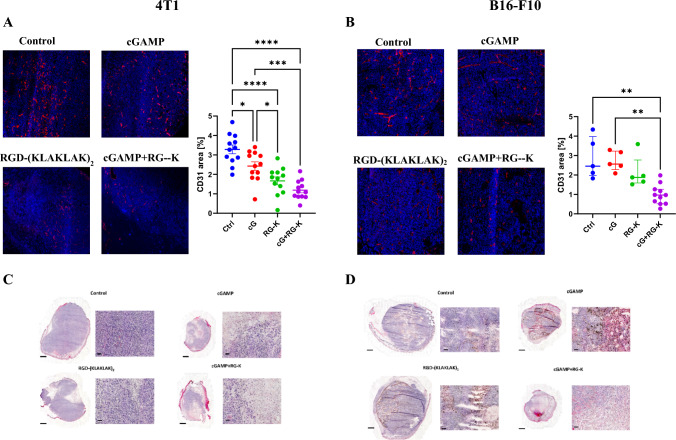
The effect of applied treatment on tumor vasculature (**A**, **B**) and structure (**C**, **D**) of 4T1 (A,C) and B16-F10 (**B**, **D**) tumors. (**A**, **B**) Tumor sections were stained with anti-CD31 antibody. CD31 positive endothelial cells (Alexa Fluor 594, red) and nuclei (DAPI, blue) were visualized using confocal microscope. Photographs were taken as a scan of 6–9 adjacent fields at 20x magnification. 1–5 scans were taken from each tumor, 5 tumors of each group were scanned. Representative photographs are shown. Percentage of tumor area covered by blood vessels was calculated using ImageJ software. Data are shown as mean ± SEM for *p < 0.05, ***p < 0.001, ****p < 0.0001 by ANOVA with post-hoc Tukey. **C**, **D** Tumors sections were stained with hematoxylin and eosin. Whole tumor sections were scanned using PANNORAMIC 250 Flash III DX scanner. Tumor tissue was visualized under 1 × and 20x magnification to observe changes in tissue structure after treatment. The scale bar is 1 mm for whole tumors pictures (1x magnification) and 50 µm in 20x magnification

Both cGAMP and RGD-(KLAKLAK)_2_ are considered as anti-vascular agents. Since we have observed different level of integrin β_3_ subunit and STING protein in both tumors models we have verified the effect of RGD-KLAK and cGAMP alone or in combination on tumor blood vessels destruction. In 4T1 tumors both agents caused significant reduction in the tumor blood vessels density, in the cGAMP group the area covered by blood vessels was nearly 1.5-times smaller and in the RGD-(KLAKLAK)_2_ group almost 2-times smaller (Fig. 3A). In both models, RGD-(KLAKLAK)_2_ caused more severe damage to the tumor vasculature than cGAMP (Fig. 3A, B). However, only the combination of these two agents led to the greatest reduction in the density of tumor vasculature (in 4T1 and B16-F10 nearly 3-times reduction of area covered by blood vessels) (Fig. 3A, B). After the therapy, we noted that the administration of an anti-vascular compound caused the appearance of necrosis areas in tumors in both tumor models (Fig. 3C, D). We observed destruction of blood vessels and formation of necrotic areas around them. We recognized that administration of the STING agonist-cGAMP resulted in the appearance of necrosis in tumors and infiltration of immune system cells. We observed remaining areas of live cancer cells ("islands") in the tumors. Although, in melanoma tumors, these areas were larger than in breast cancer tumors. In the combination therapy, more extensive areas of cancer tissue were destroyed than in either monotherapy separately, and the remaining areas of living cells were smaller.

### The effect of combination therapy on innate immune response

**Fig. 4:**
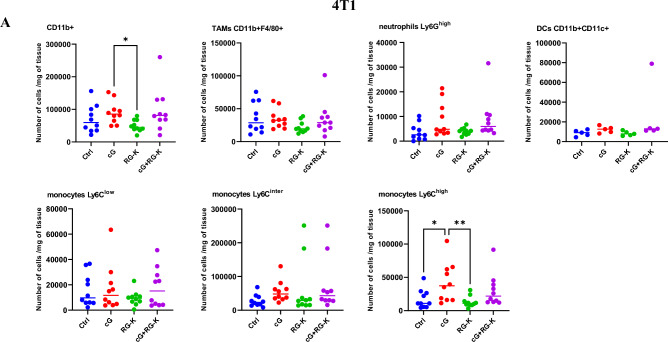

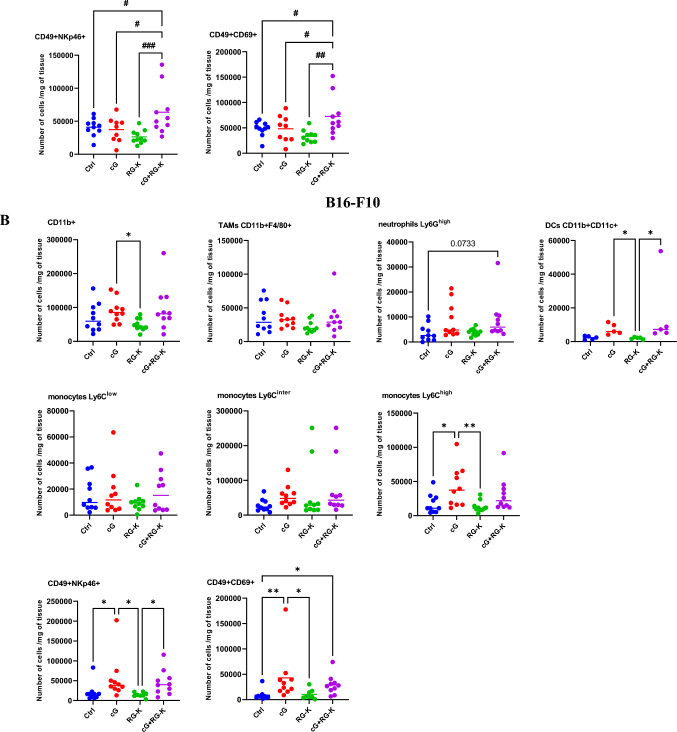
Innate immune cells infiltration in 4T1 (**A**) and B16-F10 (**B**) tumor microenvironment following applied treatments. At the end of the therapy (as depicted on therapy scheme on Fig. 2A) mice were sacrificed and tumors were collected for flow cytometry. Tumors were digested with 500U/mL of collagenase II and single cells suspension was obtained. Total myeloid cells population was gated as alive DAPI^−^CD11b^+^ cells, TAMs as: DAPI^−^CD11b^+^ F4/80^+^ cells, neutrophils as: DAPI^−^CD11b^+^CD11c^−^Ly6C^−^Ly6G^+^, dendritic cells as: DAPI^−^CD11b^+^CD11c^+^Ly6G^−^, pro-inflammatory monocytes as: DAPI^−^CD11b^+^CD11c^−^Ly6G^−^Ly6C^+^, activated NK cells as: CD45^+^, CD49b^+^, NKp46^+^/CD69^+^. The number of cells expressing specific antigens from obtained total single cell suspensions were calculated per 1 mg of tumor tissue. Data are shown as individual values with the median indicated. Results from two independent experiments are shown, *n* = 5 for each group. *p < 0.05 **p < 0.01 by Kruskal–Wallis test with Dunn's multiple comparison post hoc test, #*p* < 0.05 ##*p* < 0.01 ###*p* < 0.001 by ANOVA with post-hoc Fisher’s LSD test

We have evaluated the induction of innate immune response following applied treatments and assessed the level of tumor-infiltrating monocytes, macrophages, neutrophils, dendritic cells and NK cells at the end of the therapy. In 4T1 tumors the level of innate immune cells, besides NK cells, within the tumor microenvironment remained almost unchanged regardless of the treatment used. We have observed a slight increase in the total population of myeloid cells (CD11b^+^) and pro-inflammatory monocytes (CD11b^+^Ly6C^hi^) in groups of mice receiving cGAMP as monotherapy or in combination with RGD-(KLAKLAK)_2_. We noted a significant influx of NK cells expressing NKp46 and CD69 activating receptors following combination therapy. The number of activated NK cells was 1.5-fold higher when compared with control group (Fig. 4A). In B16-F10 tumors infiltration of innate immune cells was stronger following cGAMP administration as monotherapy or in their combination with anti-vascular peptide. We have observed increase of the overall population of CD11b^+^ myeloid cells, as well as Ly6G^hi^ neutrophils and dendritic cells CD11b^+^CD11c^+^. Similarly to 4T1 tumors the number of pro-inflammatory monocytes (CD11b^+^Ly6C^hi^) was also increased. The number of activated NK cells (NKp46^+^ and CD69^+^) in melanoma was elevated equally in groups of mice receiving cGAMP alone or in combination with RGD-(KLAKLAK)_2_ and was 2-times higher when compared with control group (Fig. 4B).

### The effect of combination therapy on CD8^+^ T cells infiltration

**Fig. 5:**
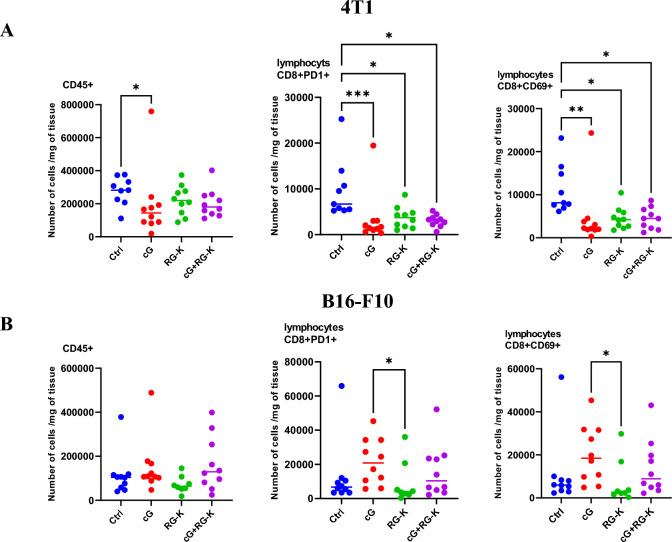
Infiltration of leukocytes and CD8^+^ T cells in 4T1 (**A**) and B16-F10 (**B**) tumor microenvironment following applied treatments. At the end of the therapy (as depicted on therapy scheme on Fig. 2A) mice were sacrificed and tumors were collected for flow cytometry analysis. Tumors were digested with 500U/mL of collagenase II and single cells suspension was obtained. The percentage of CD8^+^PD-1^+^ and CD8^+^CD69^+^ subpopulation of T lymphocytes was determined from alive DAPI^−^CD45^+^ leukocytes gate. The number of CD8^+^ T cells expressing PD-1^+^, CD69^+^ receptors were calculated per 1 mg of tumor tissue. Results from two independent experiments are shown, *n* = 5 for each group. Data are shown as individual values with the median indicated. *p < 0.05, **p < 0.01, *** *p* < 0.001 by Kruskal–Wallis test with Dunn's multiple comparison post hoc test

We have assessed the overall level of tumor-infiltrating leukocytes (CD45^+^) and the level of “exhausted” CD8^+^ T cells expressing PD-1 inhibitory receptors as well as activation marker CD69 within the TME following applied treatment. In 4T1 tumors, regardless of the therapy used, the number of leukocytes decreased by half. Each therapy resulted in reduction of exhausted CD8^+^ T cells within tumor. The number of tumor-infiltrating CD8^+^PD-1^+^ T cells was approximately 2-times lower following each of the applied treatment. The number of CD8^+^CD69^+^ activated T cells within the TME was significantly decreased in all treatment groups and was 3-times smaller than in control group (Fig. 5A). In B16-F10 tumors, treatment did not influence the total number of tumor-infiltrating leukocytes. However, unlike breast carcinoma therapy, we have observed a significant increase of CD8^+^ T cells of exhausted phenotype (CD8^+^PD-1^+^) following cGAMP administration. The increase was greater in the group that received cGAMP alone than in the group that received its combination with an anti-vascular agent. Combination with RGD-(KLAKLAK)_2_ did not further enhance the number of tumor-infiltrating CD8^+^ T cells compared with control tumors. We did not observe any statistically significant increase in the number of activated lymphocytes (CD8^+^CD69^+^) (Fig. 5B).

## Materials and methods

### Cell lines

Murine melanoma B16-F10 and 4T1 breast carcinoma (ATCC, Manassas, WV, USA) cell lines were cultured in RPMI 1640 (Thermo Fisher Scientific) supplemented with 10% heat-inactivated fetal bovine serum (EURx) and antibiotics (1% penicillin–streptomycin, Thermo Fisher Scientific).

### Therapeutic agents

Cyclic guanosine monophosphate-adenosine monophosphate (cyclic GMP-AMP, 2′3′-cGAMP, #vac-nacga23, Invivogen) was injected intratumorally in a dose 5 µg/mouse (in 100 µl PBS¯). RGD-(KLAKLAK)_2_ (purity 95.1%, GenScript) was injected intratumorally in a dose 200 µg/mouse (in 100 µl PBS¯).

### Animals, ethics and therapy

6 to 8-weeks-old mice (C57BL/6NCrl and BALB/c, Charles River Breeding Laboratories) were housed in a pathogen-free facility in SPF standard in a HEPA-filtered system. The animals received a total pathogen free standard diet and water ad libitum throughout the whole study. This study was carried out in strict accordance with the recommendations in the Guide for the Care and Use of Laboratory Animals of the National Institutes of Health. Studies on mice were performed according to the protocols approved by the Local Ethics Committee for Animal Experiments in Katowice (Permit Number: 22/2021). C57BL/6NCrl or BALB/c mice were injected subcutaneously into lower flank with 2 × 10^5^ B16-F10 cells or 2 × 10^5^ 4T1 cells in 100µL PBS¯, respectively. Tumors were measured with calipers every one or two days and volumes were determined using the formula: volume = width^2^ × length × 0.52. Mice with well-developed tumors were divided into four treatment groups: Control, cGAMP, RGD-(KLAKLAK)_2,_ cGAMP + RGD-(KLAKLAK)_2_. Animals received were treated according to group allocation: the control group left untreated with therapeutic agents, the cGAMP group with two doses of cGAMP in a two-day interval, the RGD-(KLAKLAK)_2_ group two doses of RGD-(KLAKLAK)_2_ in a two-day interval, the cGAMP + RGD-(KLAKLAK)_2_ group with two doses of cGAMP and RGD-(KLAKLAK)_2_ in a two-day interval with a one-day shift. The schedule of therapeutic agents administration is shown in Fig. 2.

### Western blot analysis

The cells were lysed with the IP buffer supplemented with protease (Thermo Fisher Scientific) and phosphatase inhibitors (Merck). Lysates were separated by SDS-PAGE electro-transferred onto PVDF membranes. The membranes were blocked and then incubated with the following primary antibodies: anti-Integrin β_3_ (clone: D7X3P, Cell Signaling Technology) and anti-β-actin (clone: AC-15, Merck). HRP-conjugated secondary antibodies were detected by chemiluminescence (Thermo Fisher Scientific).

### Immunofluorescence staining

Tumors were embedded in OCT, frozen in liquid nitrogen and sectioned into 5 µm slices. Blood vessels were stained with polyclonal anti-CD31 antibody (Abcam) and subsequently with Alexa Fluor 594 conjugated secondary antibody (Abcam). Microscopic observations were performed using a LSM710 confocal microscope. The obtained confocal images were analyzed with ImageJ 1.48v (National Institutes of Health) and the results were expressed as the percentage of area (%).

### Immunohistochemical staining

Tumors sections were fixed with 4% PFA and quenched with 0.3% H_2_O_2_. HIER was performed using antigen unmasking solution (H-3300, Vector Laboratories). Tumor slices were blocked with ultravision protein block (Epredia) and then incubated with primary anti-Integrin β3 antibody (clone: D7X3P, Cell Signaling Technology). DAB (Vector Laboratories) was used to visualize antigen. The analysis of the specimens was conducted using the Nikon Eclipse 80i microscope.

### Flow cytometry analysis

Tumors were digested with collagenase II solution (500U/ml; Gibco BRL). Red blood cells were lysed using ACK solution (Lonza). The obtained cell suspension was filtered using 70-μm and 40-μm cell strainers. Mononuclear cells were selected by centrifugation with Histopaque-1077 gradient (Merck). The isolated cells were blocked with anti-mouse CD16/32 (clone: 93) antibody (BioLegend) and stained with following antibodies: anti-CD45 (clone: 30-F11), anti-CD8 (clone: 56–6.7), anti-CD69 (clone: H1.2F3), anti-PD-1 (clone: 29F.1A12), anti-CD49b (clone: DX5), anti-NKp46 (clone: 29A1.4), CD11b (clone: M1/70), anti-F4/80 (clone: BM8), CD11c (clone: N418), Ly6C (clone: HK1.4), Ly6G (clone: 1A8) (BioLegend). Dead cells were stained by using the viability dye DAPI (Merck). In flow cytometric analyzes (BD FACSCanto II), gates dividing negative from positive cells were based on fluorescence minus one (FMO) controls.

### Statistics

The normality of distribution was verified by the Shapiro-Wilk test. The homogeneity of variance was verified by the Levene test or the Brown-Forsythe test. For variables meeting the conditions of parametric tests, analysis of variance (ANOVA) with post-hoc Tukey HSD or LSD tests was performed. Those variables are shown as mean ± SEM. For variables not meeting the conditions of parametric tests, the Kruskal-Wallis test was performed and variables are presented as median ± interquartile. All statistical comparisons were performed using Statistica 13 and GraphPad Prism V.8.0 software products.

## Discussion

Activation of cGAS-STING signaling pathway with STING agonists results in anti-tumor immune response mediated by innate immunity stimulation. Preclinical studies have shown robust anti-tumor response mediated by production of type I interferons which are known to enhance antigen presentation, augment B cell antibody production, and to amplify the function of dendritic cell and T cell population [6]. Murine models also revealed important role of NK cells in the anti-tumor activity following cGAS-STING pathway activation [5, 7]. Although STING agonists presented potential in cancer therapies, tumor resistance to STING monotherapy has emerged in clinical trials [8, 9]. Therefore, the evaluation of optimal drug combinations is needed to potentiate STING agonists in clinical use.

In our recent work [5] we have shown that intratumoral STING protein level greatly influence the outcome of therapy exploiting STING agonist, including immune cell contribution and immune checkpoint inhibitors (ICIs) responsiveness. We have observed growth inhibition of tumors characterized by high levels of STING protein, while in the case of tumors with low STING protein level, only the combination with the anti-vascular agent – combretastatin CA4P, resulted in local control of tumor growth. In the present study, we combined activation of STING pathway with a recombinant two-domain RGD-(KLAKLAK)_2_ peptide causing targeted apoptosis of cells with expression of α_v_β_3_ integrin receptors. RGD motif (tripeptide (Arg-Gly-Asp)) enables effector domain motif (KLAKLAK)_2_ to internalize into the target cell. (KLAKLAK)_2_ is one of the antimicrobial peptides, which have become promising agents for the treatment of cancer by inducing apoptosis though their preferential binding and disruption of negatively charged membranes, such as the mitochondrial membrane. Positively charged (KLAKLAK)_2_ due to its polarity cannot cross the cellular membrane therefore relies on the use of targeted delivery [10]. Thus, a ‘homing’ peptide sequence is needed improving cell penetration and the therapeutic efficacy of (KLAKLAK)_2_ motif. (KLAKLAK)_2_ based peptides have shown anti-tumor potential in among others gastric cancer [11], glioma [12, 13], melanoma [14] and breast carcinoma preclinical studies [15].

The target for RGD motif, α_v_β_3_ integrin, is overexpressed not only on the luminal side of tumor endothelium but is also expressed in some types of cancer cells. Therefore, RGD-(KLAKLAK)_2_ peptide is supposed to target two separate tumor compartments formed by tumor vasculature and neoplastic cells. Similarly to Smolarczyk et al. [14] we found expression of β_3_ subunit of the receptor on B16-F10 murine melanoma cells. This subunit was also detected on tumor blood vessels in melanoma. In 4T1 breast cancer cells the expression of β_3_ subunit was much lower and its expression in breast carcinoma tissue was limited to endothelial cells (Fig. 1A, B, C). However, 4T1 tumors responded better to RGD-(KLAKLAK)_2_ monotherapy than B16-F10 tumors, despite lower expression of target receptors. This might be due to a higher density of blood vessels in the 4T1 tumor and larger areas of necrosis resulting from intratumoral peptide administration. B16-F10 tumors are characterized by weaker vascularization, and only half of neoplastic cells express α_v_β_3_ integrin receptors. Therefore, targeting endothelial cells may kill more tumor cells than drugs targeting only tumor cells, even the inaccessible ones [16, 17]. This proves the validity of using anti-vascular therapy in the effective elimination of cancer cells.

The growth of tumor depends on nutrients and oxygen supplied by tumor blood vessels, which gradually expand as the tumor grows. Consequently, anti-vascular treatment has emerged as an important strategy to directly treat cancer by cutting off the supply nutrients and oxygen, resulting in inhibition of tumor growth. Both agents, cGAMP and RGD-(KLAKLAK)_2_ are considered as vascular disrupting agents. They indeed disrupt tumor vasculature, however we showed a greater potential for RGD-(KLAKLAK)_2_ to destroy blood vessels than cGAMP. Nevertheless, a combination of two factors caused the most severe destruction of tumor vasculature. Precisely, in 4T1 tumor both cGAMP and RGD-(KLAKLAK)_2_ monotherapies resulted in similar tumor growth inhibition, whereas in B16-F10 tumors cGAMP monotherapy was significantly more effective than peptide monotherapy. Nevertheless, after initial tumor reduction, we observed re-growth of the tumor, possibly due to viable rim cells remaining at the periphery of the tumor, nourished by surrounding unaffected blood vessels [4]. It is consistent with other papers claiming that anti-vascular treatment used as monotherapy cause moderate tumor growth inhibition [18–20]. Therefore, inducing immune response toward remaining viable rim cells seems rationale therapeutic strategy.

It has been shown that cGAS-STING pathway activates both innate and adaptive immune response [21], thereby facilitates the transformation of a "cold" tumor immune environment into a "hot" tumor-immune microenvironment [22]. Cold tumors are characterized by limited immune cell infiltration and a weak immune response within the tumor microenvironment [22, 23], while hot tumors have abundant immune cell infiltration and an active immune response [22]. Both tumor models used in our study, are characterized by "cold" tumor immune environment. We have noticed an elevated level of myeloid cells population in both melanoma and breast carcinoma tumors following cGAMP administration alone or in combination with RGD-(KLAKLAK)_2_. cGAMP administration caused infiltration of pro-inflammatory Ly6C^high^ monocytes in both tumor models. Ly6C^high^ cells are enriched in the acute phase of inflammatory response and show a proinflammatory ability. They exert proinflammatory functions mediated by secreted inflammatory factors, including TNF, IL-1β, and TGF-β [24]. Macrophages phenotypes have been described as continuous spectrum-M1/M2 paradigm. However, researchers have begun to focus on the Ly6C^hi/lo^ phenotype outside the M1/M2 classification. Ly6C^hi/lo^ and M1/M2 macrophages to some extent overlap the expression profile. Ly6C^hi^ macrophages express some signature of M1 markers, including TNF, iNOS and IFN-γ, and M2 markers, including TGF-β and IL-10. Ly6C^lo^ macrophages express some M2-specific markers, including CD206 and CD301 [24]. The main cell population that changes the most after therapy with the use of cGAMP and anti-vascular peptide combination is the population of NK cells. The level of activated NK (CD49b^+^NKp46^+^; CD49b^+^CD69^+^) cells was significantly increased following combination therapy in breast tumors, while in melanoma tumors their level was elevated both following cGAMP administration and in its combination with RGD-(KLAKLAK)_2_. NK cell–mediated tumor rejection induced by therapeutic applications of STING agonist was demonstrated in numerous tumor models, including both MHC I–deficient and MHC I–sufficient tumor models [25–27]. Such agonists enhanced NK cell activation, cytotoxicity, and anti-tumor effects inducing type I interferons (IFN) [28]. Moreover, NK cell–mediated anti-tumor response can be successfully elicited in CD8^+^ T cell-resistant tumors. Wolf et al. showed that combination of STING agonist with IL-2 improves the efficacy of tumor growth inhibition and that the effect is associated mainly with NK cell activation, not with CD8^+^ T cells expansion. Only depletion of NK cells and not CD8^+^ or CD4^+^ T lymphocytes abrogated the therapeutic effect of the combination [7].

Increased infiltration of CD8^+^ T cells within the TME in such therapy was only present in melanoma tumors following cGAMP administration. However, infiltrating CD8^+^ T cells were of exhausted phenotype characterized by PD-1 expression. Activation of cGAS-STING pathway may therefore restore tumor’s immune checkpoint responsiveness and further combination with anti-PD-1 treatment should bring beneficial therapeutic effect [5]. Despite proven enhanced anti-tumor CD8^+^ T cells response following STING activation [29–31], we observed the drop in the number of CD8^+^ T cells within breast carcinoma TME following applied treatments which requires further explanation. Our observations indicate that in CD8^+^ T cell-resistant tumors, activation of NK cells may lead to inhibition of tumor growth and in the case of such tumors, treatment with immune checkpoint inhibitors could be ineffective, which is consistent with our previous results that show no additional benefits of using the PD-1 inhibitor in tumors with suppressed CD8^+^PD-1^+^ T cell level [5]. Therefore, further studies to develop NK activating therapeutic strategy are needed to combat CD8^+^ T cell–resistant cancers.

Taken together, the results may indicate that activation of cGAS-STING pathway is sufficient to initiate the anti-tumor immune response and to modulate the TME of “cold” tumors with initially high levels of STING protein. Whereas in tumors in which STING protein level is low, its activation does not lead to a strong, adaptive immune response. Therefore, in those tumors the combination with additional therapeutic agent, such as anti-vascular treatment, is rational to enhance the anti-cancer therapeutic effect.

## Data Availability

The datasets generated during and/or analyzed during the current study are available from the corresponding author on reasonable request.
